# Dynamics between Sleep Pattern and Behavioral and Psychological Symptoms of Dementia (BPSD)

**DOI:** 10.1093/geroni/igaf122.2918

**Published:** 2025-12-31

**Authors:** Eun Kyo Kim, Sinwoo Hwang, Minhee Yang, Eunhee Cho, Jungwon Cho, Chang Gi Park

**Affiliations:** Yonsei University, Seoul, Republic of Korea; Yonsei University, Seoul, Republic of Korea; Yonsei University, Seoul, Republic of Korea; Yonsei University, Seoul, Republic of Korea; Yonsei University, Seoul, Republic of Korea; University of Illinois Chicago, Chicago, Illinois, United States

## Abstract

A higher prevalence of behavioral and psychological symptoms of dementia (BPSD) is associated with a higher care burden in caregivers and increased mortality in people living with dementia. However, from the perspective that unmet needs trigger BPSD, there is a lack of time series analysis on whether sleep disturbances can predict the occurrence of BPSD the following day. In this study, older adults with dementia wore an actigraphy device on their wrists continuously for two weeks for sleep data, and caregivers recorded BPSD in a daily symptom diary for two weeks. The panel vector autoregression model analyzed data from 154 older adults living with dementia and their caregivers and found that the previous day’s awakenings during sleep had a significant effect on irritability and appetite or eating disorders. Conversely, some symptoms of BPSD, including delusions, had significant effects on the next day’s sleep patterns, resulting in increases in total sleep time, number of awakenings, and mean awakening length. In particular, irritability among BPSD was found to have a bidirectional causality with number of awakenings. This study demonstrates that the relationship between BPSD and sleep patterns is dynamic, characterized by a vicious cycle. Consequently, early intervention to alleviate symptoms is imperative, and strategies to enhance sleep quality should be prioritized during the initial stages when sleep patterns are disturbed.

